# Effectiveness of Artificial Intelligence–Based Nursing Interventions for Chronic Illness Care: Umbrella Review

**DOI:** 10.2196/97905

**Published:** 2026-07-15

**Authors:** Jee Young Joo, Megan Liu, Youngwoo Cho, Hyungbin Cho

**Affiliations:** 1 College of Nursing, Gachon University, 191 Hambangmoe-ro, Yeonsu-gu, Medical Science Building, Room 902, Incheon, 21936, Republic of Korea, +82 32 820 4232; 2 School of Gerontology and Long-Term Care, College of Nursing, Taipei Medical University, Taipei, Taiwan

**Keywords:** artificial intelligence, chronic illness, nursing intervention, systematic review, umbrella review

## Abstract

**Background:**

Artificial intelligence (AI)–based nursing interventions are increasingly being used to manage chronic illnesses; however, their definitive impact on clinical outcomes remains inconclusive, necessitating a comprehensive evidence synthesis.

**Objective:**

This umbrella review aimed to synthesize the evidence regarding the effectiveness of AI-based nursing interventions for chronic illness care and their subsequent impact on health care outcomes in clinical settings.

**Methods:**

We conducted an umbrella review and prospectively registered the protocol. A systematic search of 5 electronic databases (PubMed, CINAHL, Cochrane Library, Scopus, and Web of Science) was performed to identify systematic reviews and meta-analyses published in English between 2021 and 2025. The methodological quality of the included studies was evaluated using the Joanna Briggs Institute (JBI) Critical Appraisal Checklist.

**Results:**

Eight high-quality systematic reviews were included, with machine learning identified as the predominant technology. Three primary outcome domains emerged: predictive, psychosocial, and hospital utilization. Due to measurement heterogeneity, the results were synthesized narratively. Our findings demonstrated that AI-based nursing interventions are effective in predicting adverse clinical events, unplanned hospital utilization, and health care costs. However, evidence regarding psychosocial outcomes remains insufficient.

**Conclusions:**

This review provides systematic evidence supporting the utility of AI in chronic illness management, particularly for improving predictive and utilization outcomes. These findings offer actionable insights for nursing leaders to integrate AI into clinical practice and education. Future research should prioritize rigorous empirical designs to further strengthen the evidence base for AI-driven nursing care.

## Introduction

### Background

In recent years, the evolution of artificial intelligence (AI) has led to major transformations in the nursing domain. The World Health Organization (WHO) announced that health care systems should use AI technologies for patient-centered and continuous care [1]. The International Council of Nurses (ICN) has highlighted that the adoption of AI is an innovative approach to health care and a potentially beneficial tool for providing nursing care to patients to enhance global health and wellness [2]. Ultimately, AI technologies adopted in health care systems have been integrated into nursing research, practice, and health care systems.

Globally, chronic illnesses have been a serious public health problem for decades. Referred to as “the Silent Pandemic,” chronic illnesses account for approximately 74% of global deaths and pose challenges to global health systems and the economy [1]. Individuals with chronic illnesses experience multiple comorbidities, require continuous care from nurses, and experience recurrent planned or unplanned hospital visits. Evidence from nursing research has shown that interventions by nursing professionals improve chronic illness care. Nurses are key health care professionals who provide patient-centered and effective coordinated care to improve chronic illness outcomes [3,4].

With the evolution of AI in nursing, extensive efforts have been made to apply AI technologies and algorithms. Specific AI techniques in nursing include machine learning (ML), deep learning (DL), robotics, decision support systems, and natural language processing [5,6]. AI algorithms are computational procedures that enable computers to execute specific tasks by following predefined rules and processing data. Common AI algorithms include decision trees, logistic regression, random forests, neural networks, and Naïve Bayes [7]. AI techniques and algorithms have been used to predict patient conditions and risks, support disease diagnosis, and provide personalized care [8,9].

Several reviews have examined AI-based health care interventions and their impact on patient care [10-12]. Previous evidence has also explored the application of AI in nursing research, practice, and education [5,13,14]. In addition, reviews focusing on AI-based nursing interventions for chronic illness care have been conducted [15-17]. However, the findings regarding the effectiveness of AI-based nursing interventions remain inconsistent because of varied outcome measures and a lack of consensus regarding their clinical impact. This evidence gap may hinder the advancement and implementation of AI-supported nursing care for chronic illnesses. Therefore, a comprehensive synthesis of current evidence is needed. Accordingly, this umbrella review aimed to identify and evaluate the effectiveness of AI-based nursing interventions for chronic illnesses and their impact on health care outcomes in clinical settings.

### Objective

This study addressed two research aims: (1) to identify nursing interventions in health care settings that apply AI in caring for chronic illnesses to influence health care outcomes and (2) to synthesize and evaluate the impacts of AI-based nursing interventions on chronic illnesses.

## Methods

### Study Design

This study followed the umbrella review methodology developed using the Joanna Briggs Institute (JBI) guidelines [18]. An umbrella review is an approach that compiles the best evidence from existing published systematic reviews (SRs) to obtain a single comprehensive review [18]. This umbrella review methodology is useful for aggregating and expanding recent issues and for highlighting synthesized evidence for future directions in health care research [18]. This umbrella review was conducted using the PICO (Population, Intervention, Comparison, and Outcome) framework to define the eligibility criteria and guide paper selection [19]. The review process adhered to the PRISMA (Preferred Reporting Items for Systematic Reviews and Meta-Analyses) guidelines (see ). The PICO framework was considered appropriate because the review sought to capture a broad range of evidence regarding AI-based nursing interventions across diverse intervention designs, health care settings, and patient outcomes. This review has been registered with the PROSPERO (International Prospective Register of Systematic Reviews) database. No institutional review board approval was required because no humans were included in the study.

### Search Strategy

Five electronic bibliographic databases (CINAHL, Cochrane Library, PubMed, Scopus, and Web of Science) were searched to identify relevant studies. The complete search strategy for each database, including Boolean operators and keyword combinations, was developed in consultation with the research team and is provided in Table S1 of Multimedia Appendix 1 to enhance reproducibility.

The following keywords and Medical Subject Headings were used with Boolean operators (AND and OR): (“artificial intelligence” OR “AI” OR “machine learning” OR “technology”), (“systematic review” OR “meta-analysis”), and (“chronic” OR “chronic disease” OR “chronic illness”) or (“nurse” OR “nursing”). Several additional search terms were used: “prediction” or “effectiveness” or “health outcome” or “nursing research.” To capture the most current evidence regarding the rapid evolution of AI in nursing, we limited our selection to SRs published within the past 5 years. Therefore, SRs with and without meta-analyses published between January 2021 and December 2025 were included in this study. This time frame was selected because AI technologies in nursing and health care have rapidly evolved in recent years, particularly following the increased implementation of ML, conversational agents, and predictive analytics in clinical practice after 2020. Restricting this review to recent SRs enabled the inclusion of the most contemporary evidence reflecting current AI capabilities, health care infrastructure, and nursing practices. However, we acknowledge that this restriction may have excluded earlier relevant evidence and potentially introduced selection bias. Articles available ahead of print publications or as online-first publications were also included. The initial search was conducted on December 1, 2025, and a confirmatory search was conducted on February 28, 2026, to identify potentially eligible studies.

### Inclusion and Exclusion Criteria

Textbox 1 shows the inclusion and exclusion criteria. Based on the PICO framework, the Population (P) included patients with chronic illnesses (eg, diabetes, hypertension, and chronic kidney disease) who received nursing care services; the Intervention (I) was defined as the integration of AI-based technologies into nursing interventions in health care settings. The Comparison (C) included the usual care, control groups, or non–AI-based interventions. The Outcome (O) included quantitative patient outcomes. The eligible studies were SRs with or without meta-analysis that included primary empirical studies such as cross-sectional studies, quasi-experimental studies, and randomized controlled trials (RCTs).

Textbox 1. **Inclusion and exclusion criteria.**

**Inclusion criteria**

- Quantitative systematic review or systematic review with meta-analysis focused on nursing interventions (nurse-led or nurses involved and nursing scholars conducted) that explicitly applied artificial intelligence (AI)–based technologies to chronic ill patients
- Systematic reviews that targeted chronically ill patients who diagnosed diseases or admitted to hospitals or health care facilities with nursing care services
- Systematic reviews with nursing interventions (nurse-led interventions) that explicitly and strictly applied AI-based technologies to patients who may benefit
- Systematic reviews published in peer-reviewed academic journals
- Systematic reviews synthesized empirical studies as their included primary studies
- Systematic reviews published from January 2021 to December 2025 (included ahead of print)

**Exclusion criteria**

- Editorials, policy papers, commentaries, or qualitative synthesis or qualitative reviews
- Reviews that did not include patients as participants and did not report patient outcomes
- Reviews that did not use AI-powered health technologies and not mainly delivered by nurses (eg, papers that used clinical decision systems that are not based on AI are excluded)
- Reviews that were published in non–peer-reviewed academic journals
- Reviews that did not focus on nursing or nurses or were not conducted by nursing scholars
- Studies in languages other than English

In this umbrella review, AI-based nursing interventions were operationally defined as nursing-related interventions that incorporated AI technologies to support assessment, clinical decision-making, patient monitoring, communication, education, or care coordination for patients with chronic illnesses. In this review, AI-based nursing interventions refer to those that incorporate AI technologies, such as ML models, predictive analytics, or clinical decision support systems, to support nursing assessment, decision-making, monitoring, or care coordination. These interventions included nurse-led applications implemented directly by nurses or nurse practitioners (NPs), as well as AI-supported systems integrated into nursing workflows within health care settings. Studies were included if AI technologies were integrated into nursing care delivery, nursing assessment, nurse-led chronic disease management, or other nursing-related clinical workflows. However, studies focusing solely on institution-level administrative AI systems without direct relevance to nursing care delivery were excluded. This umbrella review also excluded publications that were not published in English or that indicated the use of generative AI, such as ChatGPT, in manuscript writing.

In this umbrella review, “adverse events” were operationally defined as undesirable clinical outcomes associated with chronic illness progression or health care utilization, including mortality, unplanned hospital readmission, intensive care unit (ICU) admission, and disease-related deterioration. “Complications” referred to disease-related secondary conditions such as neuropathy, nephropathy, retinopathy, or other chronic illness–related sequelae. “Hospital utilization outcomes” included hospital readmissions, emergency department visits, length of hospital stay, and health care–related costs. To improve consistency and interpretability, these terms were used consistently throughout this review.

### Study Selection

Two reviewers independently screened the titles, abstracts, and full texts based on the predefined eligibility criteria. Any disagreements regarding study inclusion were resolved through discussion and consensus among all research team members. When consensus could not be reached initially, the final decision was made after a repeated review of the full-text article and methodological criteria. To assess potential double-counting bias, overlap among the included SRs was examined using a citation matrix. The primary studies included in each SR were extracted and compared across reviews to identify overlapping studies and duplicate publications.

### Quality Appraisal of Selected SRs

For umbrella reviews, it is recommended to assess the professional and methodological quality of the included SRs using a validated checklist [18,20]. This study used the JBI Critical Appraisal Checklist for SRs [21]. This checklist is recommended for umbrella reviews and provides a structured assessment of the methodological quality of SRs. It was developed to evaluate research methodologies and address the risks of bias in included studies when conducting an umbrella review [20]. Therefore, it was considered appropriate for the methodological approach in this umbrella review. The checklist consists of 11 questions in the following domains: study aim, search strategy, inclusion and exclusion criteria, data selection and extraction, publication bias, and appropriate results. These questions have 4 response options: “yes,” “no,” “unclear,” and “not applicable.” The total sum of responses ranged from 0 to 11; if the answer was “yes,” it was scored as 1; otherwise, it was scored as 0. The JBI checklist does not provide predefined cutoff scores to classify reviews as high, moderate, or low quality. However, high scores indicate high quality and low risk of methodological bias. The first and second researchers independently evaluated the quality of the 8 selected studies using the checklist.

### Data Extraction and Synthesis

In this study, data were extracted by the primary researcher using an Excel data sheet. A standardized Excel-based extraction form was developed prior to data extraction to improve consistency and reproducibility. The form included (1) bibliographic details (authors, types and designs of reviews, authors’ nationality, and publication years), (2) aims, (3) number of primary studies used in an SR, (4) details of databases used, (5) quality assessment tools, and (6) study participants. A summary of the types of AI reported in the selected studies, the algorithms used, and the findings of common health care outcomes was also extracted onto another data sheet. The primary researcher extracted the data, and the third researcher verified the extraction.

Due to substantial heterogeneity across the included SRs in terms of AI techniques, intervention characteristics, patient populations, chronic illness types, outcome measurements, and study designs, a quantitative meta-analysis was considered inappropriate. In particular, the included reviews used diverse outcome measures and varying definitions of health care outcomes, which limited the comparability of pooled effect sizes. Therefore, a narrative synthesis approach was adopted to comprehensively summarize and interpret the findings.

## Results

### General Characteristics of Included Reviews

**Figure 1.**
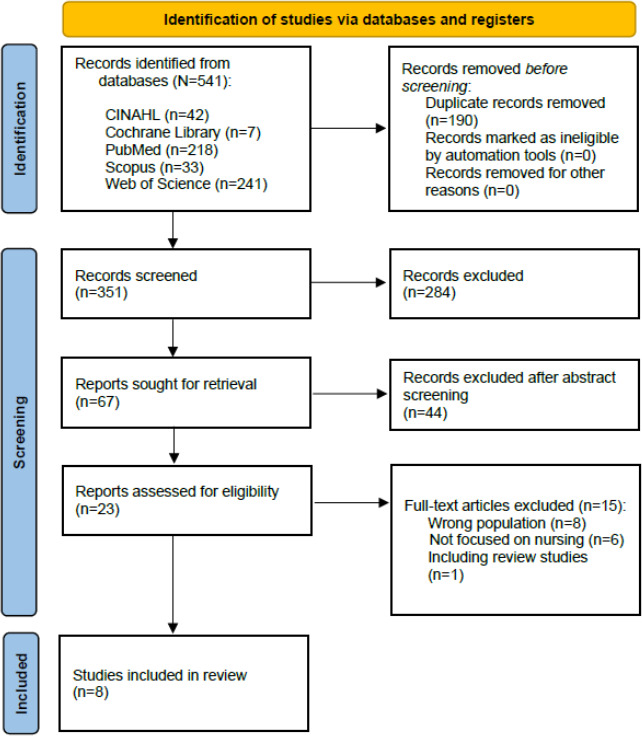
PRISMA (Preferred Reporting Items for Systematic Reviews and Meta-Analyses) 2020 flow diagram.

Figure 1 presents a summary of the search process and study selection outcomes using a PRISMA flow diagram. A total of 541 records were initially identified from the 5 electronic databases. After removing duplicate records (n=190) using EndNote X9 (Clarivate Analytics), 351 unique records remained for screening. Of these, 284 records were excluded during title screening, and 44 records were excluded after abstract screening based on the predefined eligibility criteria. Full-text eligibility assessment was conducted for the remaining 23 reports, and 15 reports were excluded because they did not meet the inclusion and exclusion criteria. Finally, 8 SRs were included in this umbrella review.

Table 1 presents the general characteristics of the 8 SRs, including the study aim, number of primary studies included, review methods, sample sizes, and year of publication. The 8 SRs were published between 2021 and 2025 [4,15-17,22-25]. These studies investigated the impact of AI-based nursing interventions on patients with chronic illnesses and analyzed their health care–related outcomes resulting from the interventions. The 8 included reviews were conducted in China (n=2), the United Kingdom, the United States, Canada, South Korea, Indonesia, and Slovenia (n=1 each), and they all followed the PRISMA guidelines . Three SRs had registered their review protocols with PROSPERO, while the rest did not report any registration. Three SRs included only RCTs [16,22,25]. Among the included SRs, only 1 SR conducted a meta-analysis [17].

The 8 SRs included a total of 150 primary study records. A citation matrix identified 7 overlapping primary studies across the reviews, corresponding to 8 duplicate publications. After accounting for overlap, 142 unique primary studies were represented in this umbrella review (Table S4 in Multimedia Appendix 1). Given the relatively small number of overlapping studies, the risk of double-counting bias was considered limited. Among these, 41 studies were RCTs, and the rest were quasi-experimental, retrospective cohort, or cross-sectional studies. Each SR included 5 to 65 primary studies published across various continents, such as North and South America, Europe, and Asia, from 2008 to 2024. Although all the SRs were published in English, the primary studies included in the reviews were published in the English, Chinese [17], or Korean [25] languages. These 2 SRs, which included primary studies in languages other than English, used translation and back-translation methods.

Based on the inclusion criteria, the participants were patients aged at least 16 years or older who had at least 1 chronic illness, such as type 2 diabetes mellitus or cancer, had been hospitalized in ICUs, or had visited the emergency department due to their chronic illness. The number of participants in primary studies ranged from 28 to 469,976.

**Table 1.** Characteristics of the included reviews (N=8).

Review (year) country	Title	Aim	Review characteristics (number of primary studies: primary study designs/review methods/PROSPERO^a^ registration/review type)	Search characteristics (DBs^b^/time frame/search strategy/number of countries of the primary studies)	Study characteristics (patients; datasets/sample size range or total number of samples/quality assessment tool of review/year of primary studies)
Bressler et al [15], (2025) United States	Leveraging AI/ML models to identify potential palliative care beneficiaries: a systematic review	To examine how ML^c^ has been used in palliative care, specifically focusing on models used to identify potential beneficiaries of palliative services among individuals with chronic and terminal illnesses	5: 1 RCT^d^, 1 quasi-experimental study, and 3 retrospective cohort studies PRISMA^e^ N/A^f^ SR^g^	4: PubMed, CINAHL, Cochrane, and Ovid No restrictions Search terms used English only publications 2 (United States 4 and Germany 1)	Patients comprising adults (aged >19 y) living with advanced illnesses 53,836 adult patients Revised Cochrane Risk-of-Bias tool for randomized studies (RoB 2.0; Sterne et al, 2019) and the Newcastle Ottawa Scale (NOS; Wells, 2021) 2018-2023
Gosak et al [16], (2024) Slovenia	AI-based prediction models for individuals at risk of multiple diabetic complications: a systematic review of the literature	To determine the effectiveness of predicting multimorbid diabetes-related complications with AI^h^-based models and determine which methods provide the best results in terms of prediction performance	11: 11 RCTs PRISMA N/A SR	4: PubMed, CINAHL, MEDLINE, and Scopus No restrictions Search terms used English language publications N/A	T2DM^i^ patients; datasets from electronic health record 72 to 135,492 NR^j^ specific tool (based on inclusion and exclusion criteria) 2015-2021
Hu et al [17], (2025) China	Prognostic models for unplanned intensive care unit readmission risk prediction: a systematic review and meta-analysis based on HSROC model	To identify prognostic models for unplanned ICU^k^ readmission and compare the performance of ML models with scoring systems	65: Cohort studies PRISMA N/A SR and meta-analysis	10: CINAHL, PubMed, EMBASE, Cochrane, ProQuest, Web of Science, CNKI (Chinese), VIP (Chinese), Wan fang (Chinese), ClinicalTrial.gov No restrictions Keywords and search terms used. 17 (N/A, but English and Chinese publications)	Patients aged 16 years or older who were hospitalized in the ICU for more than 24 hours 421 to 469,976 Prediction Model Risk of Bias Assessment Tool (PROBAST) 2008-2024
Kurniawan et al [22], (2024) Indonesia	A systematic review of AI-powered chatbot intervention for managing chronic illness	To assess user satisfaction, intervention efficacy, and the specific characteristics and AI architectures of chatbot systems designed for chronic diseases	10: RCTs (10) PRISMA Yes SR	6: PubMed, CINAHL, Embase, PsycINFO, ACM Digital Library, and Scopus 2013-2021 Keywords and search terms used. English publications 7 (United States 3, France, Spain, Australia, Germany, Switzerland, Egypt)	Patients with various chronic conditions 37 to 187 RoB 2.0 tool 2013-2021
Li et al [23], (2023) Hong Kong, China	Feasibility and effectiveness of AI-driven conversational agents in health care interventions: a systematic review of randomized controlled trials	To examine the feasibility and effectiveness of conversational agent-based interventions evaluated by RCTs in the health care context, as well as to evaluate the information quality of AI-driven conversational agents	21: RCTs (21) PRISMA Yes SR	7: Scopus, PubMed, Embase, PsycINFO, Cochrane Library, Information Science & Technology, and Web of Science No restrictions Keywords and search terms used. English publications 9 (United States 11, South Korea 2, United Kingdom, France, Spain, Australia, Switzerland, Argentina, Japan)	Adult population with various chronic conditions (at least 18 y) 28 to 927 (mean=160), with a median of 82 participants RoB 2.0 tool 2013-2021
O’Connor et al [24], (2024) United Kingdom	The application and use of AI in cancer nursing: a systematic review	To (1) examine the areas of cancer nursing AI has been applied in, (2) determine how involved cancer nurses were in AI research, and (3) understand the limitations and risks of AI in cancer nursing	20: observational, cross-sectional, case-control, etc PRISMA NR SR	4: CINAHL (EBSCOhost), MEDLINE (Ovid), PubMed (Central), and PsycINFO (Ovid) 2010-2022 Keywords and search terms used. English publications 10 (China 7, United States 3, South Korea 3, Denmark, Iran, Italy, Japan, Switzerland, Taiwan, Netherlands)	Patients with cancer 33 to 12,358 NR 2011-2022
Raymond et al [4], (2022) Canada	Nurse practitioners’ involvement and experience with AI-based health technologies: a systematic review	To characterize NPs’^l^ involvement with AI-based health technologies in terms of (1) its expected impact on the clinical activities and performance of NPs and (2) its potential outcomes for NPs’ patients and for the general population	11: retrospective studies, cross-sectional, prospective, etc PRISMA NR SR	4: CINAHL, Medline, PubMed, and Scopus NR Keywords and search terms used. English publications	ED^m^, primary, or hospitals (tertiary) visited patients NR Joanna Briggs 2018-2022
Yi et al [25], (2025) South Korea	The effects of applying AI to triage in the emergency department: a systematic review of prospective studies	To identify the intervention contents of AI-based emergency patient triage systems and investigate the intervention effect of AI-based emergency patient triage systems	7: 5 RCTs and 2 quasi-experimental designs PRISMA Yes SR	7: CINAHL, PubMed, EMBASE, Cochrane, ProQuest, RISS, and KISS No restrictions Search terms used English and Korean publications 6 (Greece 2, South Korea 1, Germany 1, Iran 1, Taiwan 1, China 1)	ED visited patients 146 to 17,072 STROBE^n^ protocol 2017-2023

a PROSPERO: International Prospective Register of Systematic Reviews.

b DB: database.

c ML: machine learning.

d RCT: randomized controlled trial.

e PRISMA: Preferred Reporting Items for Systematic Reviews and Meta-Analyses.

f N/A: not applicable.

g SR: systematic review.

h AI: artificial intelligence.

i T2DM: type 2 diabetes mellitus.

j NR: not reported.

k ICU: intensive care unit.

l NP: nurse practitioners.

m ED: emergency department.

n STROBE: Strengthening the Reporting of Observational Studies in Epidemiology.

### Summary of AI-Based Nursing Interventions in the SRs

Table 2 summarizes the characteristics of AI-based nursing interventions used in the studies. Additionally, Tables S2 and S3 in Multimedia Appendix 1 summarize the characteristics of AI-based nursing interventions used in the studies. Table S2 in Multimedia Appendix 1 provides the number of studies that use different types of AI interventions. All the primary studies clearly reported the utilization of AI techniques and AI algorithms in their nursing interventions. In this study, the AI-based nursing interventions in the reviews were classified as AI techniques [26]. The main AI interventions retrieved from the studies included ML, which was used in all 8 studies, followed by DL, clinical decision support, and natural language processing. The types of AI algorithms used were the decision tree model, logistic regression, neural networks, and Naïve Bayes. Several AI algorithms, such as regressions, Naïve Bayes, and neural networks, were used as prediction models. The most frequently used AI algorithm was logistic regression (used in all 8 SRs). Two SRs reported that chatbots and conversational agents [22,23] were used in their nursing interventions, and 1 SR used real-time voice AI support [25].

**Table 2.** Summary of artificial intelligence (AI)–based nursing interventions and outcomes in the selected systematic reviews.

Review (year)	AI-powered interventions		Major findings
	Classification of AI techniques and types of AI algorithms	Comparison	
Bressler et al [15], 2025	ML^a^ DL^b^ Decision support system Natural language processing Decision tree model Logistic regression Neural network	Usual care or NR^c^	Predictions Predict mortality risk (+)^d^: accurate and high performance in predicting 6-month (0.978), 1-year (0.956), and 2-year (0.943) mortality (2 studies) Hospital utilization outcomes Hospital readmissions (+): significant decreased hospitalizations at 60 days and 90 days (2 studies) Health care–related cost (+): cost saving (2 studies) Psychosocial outcomes (+) Quality of care (+): increased care quality (1 study)
Gosak et al [16], 2022	ML DL Random forest Naïve Bayes Decision tree model Logistic regression Neural network	NR	Predictions Predict T2DM^e^ complications (+): Risk of diabetic neuropathy (7 studies) Risk of diabetic nephropathy (6 studies) Risk of diabetic retinopathy (2 studies)
Hu et al [17], 2025	ML DL Logistic regression	Usual care with scoring systems	Predictions Predict unplanned ICU^f^ readmission (+): Scoring systems: pooled sensitivities of 0.607, with pooled specificity of 0.699 ML models: pooled sensitivities of 0.711, with pooled specificity of 0.899 DL models: pooled sensitivity of 0.745, with pooled specificity of 0.709 (indicated a higher rate of false positives) Models predicting ICU readmission within 7 days: sensitivity=0.462, specificity=0.788 Models predicting ICU readmission within 30 days: sensitivity=0.789, specificity=0.835
Kurniawan et al [22], 2024	AI-powered chatbots ML Natural language processing (3 of the included studies did not specify AI models)	Usual care and education by nurse or traditional writing or counseling	Psychosocial outcomes: Anxiety (+): positive results in the IGs^g^ (3 studies; anxiety) Depression, pain: no differences between groups (3 studies; depression, pain) Participant satisfaction (+): significantly favorable and user-friendly to use (7 studies) Patient safety (–)^h^: no transparent reporting (false alarm) of patients’ adverse events (1 study)
Li et al [23], 2023	AI-driven conversational agents–based intervention (add dosage) ML Natural language processing	Usual care	Psychosocial outcomes (+): Physical activity and function (+): significant improvement in participants' physical activity and function in the IGs (3 studies) Self-efficacy (+): significantly increased self-efficacy in healthy lifestyle maintenance (weight loss; 1 study) Illness-specific knowledge and understanding (+): significant increases in illness-specific knowledge and understanding of disease information (3 studies) QoL^i^ and well-being (+): improvement of QoL and well-being (4 studies) Depression (+): significant improvements in reducing depression (5 studies)
O’Connor et al [24], 2024	AI-based decision support system ML Natural language processing Random forest Naïve Bayes Decision tree model Logistic regression Neural network	Cancer nursing care	Predictions Predict clinical issues and complications (+): 17 studies Predictions: Predict hospital length of stay (+): significant improvements (2 studies) Psychosocial outcomes (+) Quality of care (+): increased care quality (1 study)
Raymond et al [4], 2022	ML Clinical decision support system Natural language processing		Psychosocial outcomes (±)^j^ Quality of care (±): improve the quality of health care provided by NPs (8 studies), no improvement of quality of care (1 study) Patient safety (±): improve patient safety (3 studies) Hospital utilization outcomes Rehospitalization (+): significantly reduce rehospitalization (1 study) Health care–related cost (+): cost saving in hospital care (2 studies)
Yi et al [25], 2025	Natural language processing ML DL Random forest Naïve Bayes Decision tree Logistic regression Neural network	Manual care by nurses or physicians	Predictions: Triage prediction (+): By using ESI (emergency severe index), accuracy of the AI-powered model's triage prediction ranged from 80.5% to 99.1% (4 studies) IG shows 0.9% higher accuracy with a low miss-triage rate than CG^k^ (4 studies)

a ML: machine learning.

b DL: deep learning.

c NR: not reported.

d (+): significant effectiveness.

e T2DM: type 2 diabetes mellitus.

f ICU: intensive care unit.

g IG: intervention group.

h (–): no effect.

i QoL: quality of life.

j (±): inconclusive and mixed results.

k CG: control group.

Most AI-based interventions were led by nurses or NPs [22,24,25] and delivered by nurses or NPs within multidisciplinary health care settings [4,15-17]. These interventions were directly integrated into nursing workflows, including patient monitoring and education, symptom assessment, discharge planning, follow-up, and chronic illness management [4,15-17,22,24]. Some SRs incorporated AI decision support systems and predictive algorithms [16,17,25]; however, these AI technologies were implemented within supporting nursing interventions such as assessment, clinical decision-making, and care coordination in nursing practices. The primary studies were conducted in clinical care settings, such as long-term care settings, intensive care, or emergency departments in hospitals or primary care settings.

Seven SRs reported the usual or manual care group as the control groups; however, in the remaining SR [16], such information was not clearly reported. Li et al [23] specifically described the dosage of nursing interventions, which were assisted by AI-driven conversational agents, as reported in the primary studies.

### Methodological Evaluation Results

Table 3 presents the quality assessment results of the 8 SRs. The JBI Critical Appraisal Checklist tool does not have cutoff scores; however, study scores ranging from 8 to 11 are referred to as high-quality studies, indicating a low risk of bias [27]. The review by O’Connor et al [24] scored 9 because they did not clearly and adequately report the resources that were used for the study searches and the methods used to minimize data extraction errors. The other 7 SRs scored between 10 and 11, clearly indicating the methodological directions in their reviews. Thus, all the SRs indicated a low risk of methodological bias.

**Table 3.** Joanna Briggs Institute (JBI) Critical Appraisal Checklist for systematic reviews.

JBI Critical Appraisal Criteria	Bressler et al [15], 2025	Gosak et al [16], 2024	Hu et al [17], 2025	Kurniawan et al [22], 2024	Li et al [23], 2023	O’Connor et al [24], 2024	Raymond et al [4], 2022	Yi et al [25], 2025
Is the review question clearly and explicitly stated?	Yes	Yes	Yes	Yes	Yes	Yes	Yes	Yes
Were the inclusion criteria appropriate for the review question?	Yes	Yes	Yes	Yes	Yes	Yes	Yes	Yes
Was the search strategy appropriate?	Yes	Yes	Yes	Yes	Yes	Yes	Yes	Yes
Were the sources and resources used to search for studies adequate?	Yes	Yes	Yes	Yes	Yes	Unclear	Yes	Yes
Were the criteria for appraising studies appropriate?	Yes	Yes	Yes	Yes	Yes	Yes	Yes	Yes
Was critical appraisal conducted by 2 or more reviewers independently?	Yes	Yes	Yes	Yes	Yes	Yes	Yes	Yes
Were there methods to minimize errors in data extraction?	Yes	Unclear	Yes	Yes	Unclear	Unclear	Unclear	Yes
Were the methods used to combine studies appropriate?	Yes	Yes	Yes	Yes	Yes	Yes	Yes	Yes
Was the likelihood of publication bias assessed?	Yes	Yes	Yes	No	Yes	Yes	Yes	Yes
Were recommendations for policy and/or practice supported by the reported data?	Yes	Yes	Yes	Yes	Yes	Yes	Yes	Yes
Were the specific directives for new research appropriate?	Yes	Yes	Yes	Yes	Yes	Yes	Yes	Yes
Total score (quality score)	11	10	11	10	10	9	10	11

### Main Patient Outcomes

#### Overview

Among the 8 included SRs, the primary patient outcomes of AI-based nursing interventions were categorized into 3 common domains: (1) predictive outcomes (n*=*5), (2) psychosocial outcomes (n*=*5), and (3) hospital utilization outcomes (n=2; Tables S2 and S3 in Multimedia Appendix 1). Table 2 summarizes the common outcomes of these reviews. Due to the heterogeneity of the results, the findings are synthesized narratively.

#### Predictive Outcomes

In the 5 SRs that reported predictive outcomes, the predictions of adverse events, such as disease-related complication risks, mortality risks, and possible hospital utilization, were commonly reported using predictive algorithms [15-17,24,25].

Three SRs reported predicted possible complications related to diseases using AI-based nursing interventions [15,16,24]. In a high-quality review, Gosak et al [16] reported that nursing interventions applying AI techniques could predict the possible complications of type 2 diabetes mellitus. In this review, the risks of neuropathy, nephropathy, and retinopathy associated with diabetes were precisely predicted using AI prediction algorithms, such as penalized regression, random forest, and neural networks. Bressler et al [15] predicted the mortality risks of patients at 6 months, 1 year, and 2 years. These predictions enabled the early and timely detection of nurses’ care interventions. O’Connor et al [24] reported the predicted risk of complications in patients with cancer in 17 primary studies. Yi et al [25] reviewed emergency triage predictions and found that intervention groups for which AI models were applied showed 0.9% more accurate predictions in emergency triage than those shown by control groups. In addition, the accuracy of emergency triage prediction rates ranged from 80.5% to 99.1% in the 4 primary studies. Thus, the results indicated that AI-based interventions had an impact on predicting adverse outcomes in patients.

Two SRs reported predictions of unplanned hospital utilization [17,24]. In the study by O’Connor et al [24], the length of hospital stay was predicted using 2 primary studies, with a conclusion of a significant improvement in the reduction of length of hospital stay predictions. Hu et al [17] predicted ICU readmissions using pooled results from a meta-analysis of 56 primary studies. They found that ICU readmissions within 30 days showed higher sensitivity (0.789, 95% CI 0.751-0.824) and specificity (0.835, 95% CI 0.805-0.860); sensitivity and specificity within 7 days were 0.462 (95% CI 0.118-0.846) and 0.788 (95% CI 0.448-0.945), respectively. Hu et al [17] reported that using short-term expectations to predict ICU readmissions is more complex.

#### Psychosocial Outcomes

Five SRs reported patients’ psychosocial outcomes as results of AI-based nursing interventions [4,15,22-24]. In the SRs, patients’ self-reported outcomes, such as quality of care, patient safety, patient satisfaction, and depressive symptoms, were measured.

Regarding the quality of care, 2 SRs demonstrated that the quality of care increased [15,24]. However, Raymond et al [4] reported mixed results in a high-quality SR. There was an improvement in the quality of care provided by NPs in 8 primary studies; however, no improvement was reported in 1 study. The results of patient safety outcomes were mixed. In the study by Kurniawan et al [22], AI-based nursing interventions could not support patient safety. Raymond et al [4] reported improvements in patient safety in 3 primary studies. Regarding patient satisfaction outcomes, Kurniawan et al [22] reported significant differences between groups in 7 primary studies after applying AI-based nursing interventions. Two SRs that reported depression as the primary outcome of AI-based nursing interventions for chronic illnesses [4,23] reported a significant reduction in depression in their 5 included primary RCTs. However, Kurniawan et al [22] reported no differences between the intervention and control groups in 3 studies. Therefore, the impact of AI-based nursing interventions on psychosocial outcomes remains inconclusive.

#### Hospital Utilization Outcomes

Two SRs reported total hospital utilization outcomes resulting from AI-based nursing interventions for chronic illnesses [4,15]. Both SRs investigated hospital readmissions and health care–related costs, and the results were conclusive. In the review by Bressler et al [15], 60- and 90-day hospital readmissions were significantly decreased in 1 primary study. Similarly, Raymond et al [4] reported that 2 primary studies showed significant reduction in rehospitalizations. Both SRs reported cost savings in intervention groups compared with control groups in hospital-based settings [15] and hospital care, especially in emergency care settings [4]. Both SRs, which were high-quality reviews, concluded the cost-saving capabilities of AI-based nursing interventions.

## Discussion

### Principal Findings

The integration of AI techniques into patient care has been used by nurses in various health care settings and has been reported to improve patient health care outcomes. This umbrella review expands on the current inconclusive results of AI-based nursing interventions to provide robust evidence on AI-based nursing interventions for chronic illnesses. The first aim of this umbrella review was to summarize nursing interventions that applied AI in caring for patients with chronic illnesses in health care settings, which may have affected patients’ health care outcomes by analyzing individual SRs conducted in 7 countries. In this study, we synthesized the findings from 8 SRs focusing on AI-based nursing interventions for chronic illnesses that were conducted using 143 primary studies. All 8 SRs were published recently within the past 5 years, from 2021 to 2025. This study identified the types of AI techniques and algorithms used in recent evidence-based research. Regarding the AI techniques applied in nursing interventions, ML, decision-support systems, and DL, which is a subset of ML [28], were identified in the included SRs. These findings should be interpreted in the context of the specific AI modality rather than as evidence for a single homogeneous category of AI-based nursing interventions. Among the AI algorithms, logistic regression models were most frequently used in the included reviews, as 5 of the SRs focused on predicting adverse outcomes.

Based on the secondary aim of this review, we synthesized 3 primary and common patient outcomes—predictive, psychosocial, and hospital utilization outcomes. This review identified the advantages of AI-based nursing interventions in predicting adverse events and hospital utilization outcomes. According to the SRs, applying AI algorithm models to intervention groups enabled better prediction of potential risks in patients with chronic illnesses, such as complications and unplanned hospital utilizations, compared with control groups without AI assistance. Chronic illnesses are associated with multiple comorbidities and complications [29]; thus, early-detection systems using AI are important [8,30]. Consistent with previous reviews, AI-based predictive models impact disease-related risk predictions [31,32]. In this umbrella review, we found that nurses could identify the possible risks of adverse events during AI-assisted nursing interventions. Subsequently, the nurses perceived patients’ care needs with AI algorithms and proactively provided appropriate care for patients with chronic illnesses to prevent possible adverse events [16,25]. From the included SRs, we pooled data to predict unplanned ICU readmissions using the AI technique for critical care nurses [17]. The results showed that applying AI techniques affected nurses’ practice, ensuring the prioritization of high-risk patient care. However, there could be accuracy issues and an algorithm risk of bias in AI-assisted predictions [17,25]. Thus, more rigorous AI models that can support nurses’ interventions and larger datasets to critically predict chronic illness risks are required.

This umbrella review also revealed reductions in hospital readmissions and health care–related costs. Two SRs reported that AI-based nursing interventions significantly decreased hospitalization and health care costs for chronic illnesses [4,15]. The results regarding hospital utilization outcomes are supported by multidisciplinary studies on patients at high risk or receiving postoperative care [9,33]. In this review, we concluded that hospital utilization prediction was effective; however, 2 of the included SRs (4 primary studies) reported an impact on chronic illnesses. Furthermore, none of the SRs specifically compared the full economic analysis related to hospital use. More robust evidence that includes a large dataset and an elaborate analysis of the impact of AI-based nursing interventions on hospital utilization and cost analysis for chronic illness in health care settings is recommended.

The results regarding psychosocial outcomes were inconclusive. This umbrella review found mixed results on the safety, quality of care, satisfaction, or depression status among patients with chronic illness. Owing to the complex and wide range of psychological outcome measurements, SRs reported varied outcomes. One study demonstrated a false alarm regarding patients’ adverse outcomes; thus, it reported no effectiveness of AI-based interventions on patient safety [22]. Recently, some nursing reviews have explored patient safety outcomes using AI [30,34]. However, the results have been inconclusive. Therefore, more studies on clear psychosocial measures of psychological outcomes after AI-based nursing interventions are required to provide evidence.

In the included SRs, evidence regarding the effectiveness of AI-based theoretical frameworks, specifically which AI type or algorithm effectively affects patient outcomes, is limited. In evidence-based practice, it is imperative to have theoretical frameworks that can be accepted as part of AI technology in nursing. One SR by Hu et al [17] concluded that ML outperformed other algorithms in predicting chronic illness outcomes. This finding is congruent with previous reviews, indicating that ML has been the most useful AI technology for nursing interventions [5]. Recent studies have demonstrated that AI models with AI algorithms are highly accurate in predicting patient outcomes for specific diseases in nursing research [5,10]. However, in this umbrella review, none of the SRs indicated that AI algorithms outperformed others in patient outcome predictions, nor did any show that AI algorithms were mostly used to predict patient outcomes. Further empirical studies are required to identify successful implementations of AI algorithm-optimized nursing interventions that are most useful for chronic illness care provision. Furthermore, theoretical frameworks for AI, targeting chronic illness management, should be established to implement AI-based nursing interventions.

When analyzing such AI-based nursing interventions regarding patient outcomes, it is necessary to clearly and robustly include complex datasets. Moreover, it is imperative to combine and refine all nurses’ clinical notes, including texts from other health care professionals, to identify the most effective AI algorithms or types [24]. It is important to include nursing notes or unstructured texts when analyzing patient outcomes in AI-based nursing interventions for robust data analysis [4,15,17,24]. Several SRs have reported that nurses’ free-text documentation and clinicians’ notes are useful for AI-based model development and for predicting patient outcomes [4,15]. Hu et al [17] suggested that ML outperformed other methods in predicting patient outcomes; however, they used only structured datasets and ignored clinical notes. Thus, it may be insufficient to conclude that ML is a promising AI technique for nursing interventions in care provision for chronic illness. It is recommended to use both structured and unstructured data, such as nursing notes, to predict outcome measurements, along with AI-based techniques to eliminate the risk of data bias.

### Strengths and Limitations

One strength of this umbrella review is that the 8 SR results included 41 RCTs and experimental design studies, which is a little less than half of the 143 primary studies. A recent review reported that there is a dearth of experimental studies on AI-based intervention in nursing, and it may be possible to prove the effectiveness of AI on patient outcomes in nursing research [5]. In addition, this review provides evidence that elucidates the impact of AI-based nursing interventions on related patient outcomes due to the high methodological quality of SRs.

This study has a few limitations. First, we identified and synthesized 3 common patient outcomes from 8 SRs. This review identified 3 dominant patient outcomes as the impact of AI-based nursing interventions. There could be more positive outcomes and benefits of AI-based technologies. Moreover, substantial heterogeneity in AI intervention types, outcome measures, patient populations, chronic illness types, and study designs limited the feasibility of conducting a quantitative meta-analysis. Therefore, this review could only aggregate and summarize the findings narratively. More frequently, pooled results reporting patient outcomes with meta-analytical methods are desirable. Given the heterogeneity of AI technologies, intervention characteristics, and outcome measures, these findings should not be interpreted as evidence supporting the effectiveness of AI-based nursing interventions as a homogeneous entity. Second, the 8 included SRs focused only on the effectiveness of AI technologies in participants with chronic illnesses. There was limited information about the dosage of the interventions, indicating a dearth of information about the frequency or duration of AI-based interventions. Only the review by Li et al [23] reported the dosage of interventions in their primary RCTs. Thus, this umbrella review had limited information from the included SRs to perform a dosage analysis of AI techniques and nursing interventions and its impact on patient outcomes. Third, this umbrella review included SRs published in peer-reviewed journals from 5 electronic databases in the past 5 years and excluded gray literature to meet the aim of this review. Thus, some data may have been missing. Furthermore, this study may have a potential overlap of primary studies across the included SRs, which may introduce double-counting bias and affect the validity of the findings. Fourth, participants with chronic illnesses included in the SRs were recruited within health care settings, such as emergency departments or long-term care. There was limited information about their age, number of chronic diseases, or any complications. Fifth, potential publication bias could have been present in the 8 SRs, as they had the tendency to report positive results. Finally, this review included only SRs published in English.

Several challenges should also be considered when integrating AI-based technologies into nursing practice. Algorithmic bias, data quality limitations, and the limited explainability of some AI models may affect the reliability and transparency of AI-assisted clinical decision-making. In addition, interoperability with existing electronic health record systems remains a significant barrier in many health care settings. Clinician trust, ethical considerations, data privacy, and regulatory compliance must also be addressed to support the safe and effective implementation of AI-enabled nursing interventions. Future research should evaluate not only effectiveness but also implementation feasibility, acceptability, and sustainability in clinical practice.

### Implications for Nursing Practice and Policy

For frontline nurses caring for patients with chronic illnesses, the findings of this umbrella review will enable them to identify patient risks and effectively integrate AI-based techniques into clinical nursing practice. Nurses and NPs can use the results of this umbrella review as evidence for practice in chronic illness care provision. The ICN emphasizes that nurses should participate in and be educated on the development of AI-based interventions in health care systems and implement these AI technologies in their clinical practice to achieve better patient outcomes and patient safety [2]. Evidence exists on risk predictions, hospital utilization, and health care costs in AI-based nursing interventions for chronic illnesses. Thus, nurses need to explore more patient outcomes, including the investigation of inconclusive psychological outcomes of chronic illnesses. Furthermore, it is recommended that nurses should implement and lead the application of AI in clinical practice to improve patient-centered care [2].

Nursing leaders in global nursing organizations prioritize AI in nursing research, education, and practice [1,2]. The ICN has announced the importance of digital technology education in nursing and nurses’ competency in using AI systems [2]. They must continuously leverage the empowerment of the nursing workforce within an AI health care system. They require stable support in nursing research, practice, and policy development domains to continuously realize the value of AI in clinical practice.

### Conclusion

This umbrella review is the first to synthesize recent SRs examining the impact of AI-based nursing interventions on chronic illnesses in health care settings. The findings suggest that AI-based nursing interventions may have potential benefits for predictive outcomes and for reducing unplanned hospital utilization. However, evidence regarding psychosocial outcomes remains conflicting and inconclusive. These results should be interpreted cautiously due to the limited number of included reviews and the heterogeneity across studies. Therefore, further high-quality studies are needed to strengthen the identified evidence and optimize nurse-led AI-based interventions across different outcome domains in clinical practice.

## Supplementary material

10.2196/97905 Multimedia Appendix 1 Search strategies, artificial intelligence algorithms, review characteristics, outcomes, and study overlap across included systematic reviews.

## Abbreviations

AI: artificial intelligence
DL: deep learning
ICN: International Council of Nurses
ICU: intensive care unit
JBI: Joanna Briggs Institute
ML: machine learning
NP: nurse practitioner
PRISMA: Preferred Reporting Items for Systematic Reviews and Meta-Analyses
PROSPERO: International Prospective Register of Systematic Reviews
RCT: randomized controlled trial
SR: systematic review
WHO: World Health Organization
