# Supplementary material for: Effectiveness of Artificial Intelligence–Based Nursing Interventions for Chronic Illness Care: Umbrella Review
Source: JMIR Nurs. 2026 Jul 15;9:e97905. doi: 10.2196/97905 (PMC13373462; doi:10.2196/97905)
Supplement: Multimedia Appendix 1 [file nursing-v9-e97905-s001.docx]

Supplementary Table 1. Database-specific search strategies used for the literature search

Search terms for systematic review

| AI-related terms | Nursing-related terms | Chronic illness terms | Other terminology |
| --- | --- | --- | --- |
| Artificial intelligence AI Machine learning Deep learning Clinical decision support system Decision support system Predictive model Predictive analytics Natural language processing Neural network | Nurse Nursing Nurse practitioner Nursing care Nursing intervention Nursing practice | Chronic disease Chronic illness Chronic condition Long-term condition Diabetes Hypertension Chronic kidney disease | Systematic review Meta-analysis eview |

Database-specific search strategy

| Database | Full search strategy |
| --- | --- |
| CINAHL | S1 (MH "Artificial Intelligence+") OR "artificial intelligence" OR AI OR "machine learning" OR "deep learning" OR "clinical decision support system*" OR "decision support" OR "predictive model*"  S2 (MH "Chronic Disease+") OR "chronic disease*" OR "chronic illness*" OR "chronic condition*" OR diabetes OR hypertension OR "chronic kidney disease"  S3 (MH "Nursing+") OR nurse* OR nursing OR "nursing care" OR "nursing intervention*" OR "nurse practitioner*"  S4 S1 AND S2 AND S3  Limiters: English language |
| Cochrane Library | ("artificial intelligence" OR AI OR "machine learning" OR "decision support")AND  ("chronic disease" OR "chronic illness")AND  (nursing OR nurse*)AND  ("systematic review" OR "meta-analysis")  Limits: English, 2021-2025 |
| PubMed | #1 ("Artificial Intelligence"[Mesh] OR "artificial intelligence" OR AI OR "machine learning" OR "deep learning") #2 ("Chronic Disease"[Mesh] OR "chronic illness*" OR diabetes OR hypertension OR "chronic kidney disease") #3 ("Nursing"[Mesh] OR nurs*) #4 ("systematic review" OR "meta-analysis") #5 #1 AND #2 AND #3 AND #4 Filters: English, 2021-2025 |
| Scopus | "artificial intelligence" OR AI OR "machine learning" OR "decision support")  AND  ("chronic disease" OR "chronic illness") AND  (nursing OR nurse*) AND  ("systematic review" OR "meta-analysis")  Limits: English, 2021-2025 |
| Web of Science | ("artificial intelligence" OR AI OR "machine learning" OR "decision support")  AND ("chronic disease" OR "chronic illness")  AND (nursing OR nurse*)  AND (("systematic review" OR "meta-analysis")  Language: English  Timespan: 2021-2025 |

Note: CINAHL: Cumulative Index to Nursing, and Allied Health Literature; MeSH: Medical Subject Heading

Supplementary Table 2.

| Review | Types of AI algorithms | | | | | | | | | | | Type of outcome | | |
| --- | --- | --- | --- | --- | --- | --- | --- | --- | --- | --- | --- | --- | --- | --- |
|  | ML | DL | Decision support sys | Natural language processing | Decision tree model | Logistic regression | Neural network | Random Forest | Naïve Bayes | AI powered chat bots (Or conversational agents) | Unspecified | Predictive e | Hospital utilization | Psychosocial |
| Bressler et al. (2025) | X | X | X | X | X | X |  |  |  |  |  | X | X | X |
| Gosak et al. (2022) | X | X |  |  | X | X | X | X | X |  |  | X |  |  |
| Hu et al. (2025) | X | X |  |  |  | X |  |  |  |  |  | X |  |  |
| Kurniawan et al. (2024) | X |  |  | X |  |  |  |  |  | X | X |  |  | X |
| Li et al. (2023) | X |  |  | X |  |  |  |  |  | X |  |  |  | X |
| O’Connor et al. (2024) | X |  | X | X | X | X | X | X | X |  |  | X |  | X |
| Raymond et al. (2022) | X |  | X | X |  |  |  |  |  |  |  |  | X | X |
| Yi et al. (2021) | X | X |  | X | X | X | X | X | X |  |  | X |  |  |
| TOTAL | 8 | 4 | 3 | 6 | 4 | 5 | 3 | 3 | 3 | 2 | 1 | 5 | 2 | 5 |

*Note*. DL: deep learning; ML: machine learning

Supplementary Table 3.

|  | Hu et al. (2025) | Kurniawan et al. (2024) | Li et al. (2023) | O’Connor et al. (2024) | Gosak et al. (2022) | Yi et al. (2021) | Bressler et al. (2025) | Raymond et al. (2022) |
| --- | --- | --- | --- | --- | --- | --- | --- | --- |
| **Year of publication of earliest and latest study in the review** | 2008–2024 | 2013–2021 | 2013–2021 | 2011–2022 | 2015–2021 | 2017–2023 | 2018–2023 | 2018–2022 |
| **Study location** | China | Indonesia | Hong Kong | UK | Slovenia | South Korea | USA | Canada |
| **Hospital utilization outcomes** |  |  |  |  |  |  |  |  |
| Readmission |  |  |  |  |  |  |  | X |
| Costs |  |  |  |  |  |  | X | X |
| Rehospitalization |  |  |  |  |  |  | X |  |
| **Psychosocial outcomes** |  |  |  |  |  |  |  |  |
| Quality of care |  |  |  | X |  |  | X | X |
| Anxiety |  | X |  |  |  |  |  |  |
| Depression |  | X | X |  |  |  |  |  |
| Patient satisfaction |  | X |  |  |  |  |  |  |
| Patient safety |  | X |  |  |  |  |  | X |
| Physical activity and function |  |  | X |  |  |  |  |  |
| Self-efficacity |  |  | X |  |  |  |  |  |
| Illness-specific knowledge & understanding |  |  | X |  |  |  |  |  |
| Quality of life and well-being |  |  | X |  |  |  |  |  |
| **Predictions** |  |  |  |  |  |  |  |  |
| Mortality risk |  |  |  |  |  |  | X |  |
| Complications T2DM |  |  |  |  | X |  |  |  |
| Unplanned ICU readmission | X |  |  |  |  |  |  |  |
| Clinical issues and complications |  | X |  |  |  |  |  |  |
| Length of stay (LOS) |  |  | X |  |  |  |  |  |
| Triage |  |  |  |  |  | X |  |  |

Supplementary Table 4. Overlap of primary studies across the included systematic reviews: Citation matrix

| Systematic review  Primary study | Hu et al. (2025) | Kurniawan et al. (2024) | Li et al. (2023) | O’Connor et al. (2024) | Gosak et al. (2022) | Yi et al. (2021) | Bressler et al. (2025) | Raymond et al. (2022) |
| --- | --- | --- | --- | --- | --- | --- | --- | --- |
| 1. Bibault et al. (2019) |  | X | X |  |  |  |  |  |
| 1. Echeazana et al. (2021) |  | X | X |  |  |  |  |  |
| 1. Gong (2020) |  | X | X |  |  |  |  |  |
| 1. Hause-Ulrich (2020) |  | X | X |  |  |  |  |  |
| 1. Hunt (2021) |  | X | X |  |  |  |  |  |
| 1. Kim et al. (2019) |  |  |  | X | X |  |  |  |
| 1. Wang et al. (2021) |  |  |  | X | X |  |  | X |
